# (Ag)Pd-Fe_3_O_4_ Nanocomposites as Novel Catalysts for Methane Partial Oxidation at Low Temperature

**DOI:** 10.3390/nano10050988

**Published:** 2020-05-21

**Authors:** Blanca Martínez-Navarro, Ruth Sanchis, Esther Asedegbega-Nieto, Benjamín Solsona, Francisco Ivars-Barceló

**Affiliations:** 1 Departmento de Química Inorgánica y Química Técnica, Facultad de Ciencias, UNED, Paseo Senda del Rey, 9, 28040 Madrid, Spain; blancamartinez@ccia.uned.es (B.M.-N.); easedegbega@ccia.uned.es (E.A.-N.); 2 Instituto de Catálisis y Petroleoquímica (ICP-CSIC), C/Marie Curie, 2, Cantoblanco, 28049 Madrid, Spain; 3 Departmento de Ingeniería Química, Universitat de València, C/Dr. Moliner 50, Burjassot, 46100 Valencia, Spain; rut.sanchis@uv.es (R.S.); benjamin.solsona@uv.es (B.S.)

**Keywords:** methane, oxidation catalysis, formaldehyde, magnetite iron oxide, Fe_3_O_4_, heterogeneous catalysis, palladium, Pd, silver, Ag, low-temperature activity, nanocomposite, Raman, TG in air, TG in hydrogen, XRD, electron microscopy, EDS

## Abstract

Nanostructured composite materials based on noble mono-(Pd) or bi-metallic (Ag/Pd) particles supported on mixed iron oxides (II/III) with bulk magnetite structure (Fe_3_O_4_) have been developed in order to assess their potential for heterogeneous catalysis applications in methane partial oxidation. Advancing the direct transformation of methane into value-added chemicals is consensually accepted as the key to ensuring sustainable development in the forthcoming future. On the one hand, nanosized Fe_3_O_4_ particles with spherical morphology were synthesized by an aqueous-based reflux method employing different Fe (II)/Fe (III) molar ratios (2 or 4) and reflux temperatures (80, 95 or 110 °C). The solids obtained from a Fe (II)/Fe (III) nominal molar ratio of 4 showed higher specific surface areas which were also found to increase on lowering the reflux temperature. The starting 80 m^2^ g^−1^ was enhanced up to 140 m^2^ g^−1^ for the resulting optimized Fe_3_O_4_-based solid consisting of nanoparticles with a 15 nm average diameter. On the other hand, Pd or Pd-Ag were incorporated post-synthesis, by impregnation on the highest surface Fe_3_O_4_ nanostructured substrate, using 1–3 wt.% metal load range and maintaining a constant Pd:Ag ratio of 8:2 in the bimetallic sample. The prepared nanocomposite materials were investigated by different physicochemical techniques, such as X-ray diffraction, thermogravimetry (TG) in air or H_2_, as well as several compositions and structural aspects using field emission scanning and scanning transmission electron microscopy techniques coupled to energy-dispersive X-ray spectroscopy (EDS). Finally, the catalytic results from a preliminary reactivity study confirmed the potential of magnetite-supported (Ag)Pd catalysts for CH_4_ partial oxidation into formaldehyde, with low reaction rates, methane conversion starting at 200 °C, far below temperatures reported in the literature up to now; and very high selectivity to formaldehyde, above 95%, for Fe_3_O_4_ samples with 3 wt.% metal, either Pd or Pd-Ag.

## 1. Introduction

Transformation of methane into valuable compounds is one of the hardest challenges in petrochemistry. The large abundance of methane in natural gas and the recent discoveries of new shale gas reserves around the world make methane functionalization even more interesting. If partial oxidation of methane to compounds with high value, such as methanol and especially formaldehyde, could take place with high efficiency, it could be competitive against the indirect process in several steps from synthesis gas [1]. Unfortunately, no catalytic system has shown promising values neither regarding reaction rates nor selectivity at medium and high methane conversions. The main drawback is related to the high stability of the methane molecule, which presents strong methyl C–H bonds, which are not easy to activate. Thus, it is usual that this reaction takes place at high reaction temperatures so that in those conditions the partial oxidation products are quickly oxidized into carbon oxides. As in mild reaction conditions, the reaction rates are low, and in harder conditions the carbon oxide formation is predominant; an intermediate trade-off working seems to be the best option [2]. In any case, the direct oxidation of methane into methanol, formaldehyde, and other oxygenated products is still very far from being competitive for commercial implementation [2,3]. Among the catalysts employed for direct oxidation of methane to formaldehyde using molecular oxygen, those based on vanadium, molybdenum [4,5,6,7], and especially iron [3,8,9,10,11,12,13,14,15,16,17] have shown the most promising performance. Nevertheless, the yield of formaldehyde reported with these catalysts does not exceed 5% [2,4,8,18,19,20], although higher yields can be found in the literature for other catalysts. A significant example of this is the 14–17% yield of formaldehyde patented for Mo-based heteropoly acid catalysts at 600–650 °C [8], never again mentioned, with CH_4_ conversions and formaldehyde selectivity within the 20–23% and 65–84% ranges, respectively.

In the case of iron-based catalysts, bulk FePO_4_ systems and mainly iron supported on a range of siliceous materials (standard silica, MCM-41, SBA-15, and others) have been studied in this reaction [10,14,15,16,17,21]. Although it is accepted that iron sites must not be highly aggregated, otherwise formaldehyde readily decomposes, there is not a wide consensus about the exact nature of the iron active and selective species. It has been proposed that isolated tetrahedral Fe^3+^ sites are the most selective sites [22] whereas other authors have suggested that Fe with higher aggregation (2D FeOx oligonuclear sites) is more effective than isolated Fe^3+^ sites [16]. In any case, the reaction temperatures employed in these articles exceed 400 °C and are often over 500 °C [3,8,12]. Interestingly, recent work by Zhao et al. showed that iron species supported on different zeolites (ZSM-5, beta, and ferrierite) activate methane at 350 °C using N_2_O as an oxidant, reaching methane conversions until 3%, with the formation of different partial oxidation products (methanol, formaldehyde, dimethyl ether) but also carbon oxides, obtaining up to 10% selectivity to formaldehyde [9]. It must be mentioned that the transformation of methane at room temperature has also been studied but with no competitive results or using reaction conditions not viable for commercial applications [23,24]. In this vein, the direct transformation of methane into formaldehyde has been interestingly demonstrated to be possible at room temperature using gaseous [Al_2_O_3_]^+^ clusters, although with low rates [25].

On the other hand, palladium catalysts are, together with platinum ones, usually employed in the total oxidation of methane into carbon dioxide [26,27,28,29], a very much applied reaction from an environmental viewpoint. Thus, palladium sites can activate methane at low temperatures and high conversions can be easily reached, although the methane transformation is directed towards a total combustion product of limited interest. These works mainly pay attention to the light-off curves in order to see the temperatures required for all methane to be converted and to follow the stability with the time on stream. However, due to the massive CO_2_ formation, no special interest has been paid to see what takes place at low methane conversions.

Within this context, the aim of the current work is to rationally design and develop catalytic systems with potential for the partial oxidation of methane at ambient pressure and relatively mild reaction temperatures. For such a purpose, we have drawn inspiration from materials, chemical species, and results, all known from the scientific literature as well as from our own experience. Moreover, we kept the premises of using simple preparation methods and raw materials not especially expensive, in order to develop a multifunctional catalyst which was not specifically reported for partial oxidation of methane. Thus, Fe_3_O_4_-based nanostructures supporting, in turn, smaller Pd nanoparticles, were the catalytic system selected and developed. To complement this, a systematic study on some synthesis parameters in order to improve the surface area of the iron oxide substrate without using sophisticated methods was successfully performed. An additional purpose was also to prove the use of Ag as a dopant to tune the high reactivity of Pd toward moderate catalytic behaviors rather than its usual propensity for methane combustion [30,31].

The structural and elemental compositions, textural properties, morphology, and reactivity against reduction and oxidation for the developed (Ag)Pd-Fe_3_O_4_ nanocomposites were investigated by several physicochemical techniques, as well as their catalytic properties for methane partial oxidation at temperatures not higher than 250 °C. Promising results were achieved from the preliminary catalytic screening which will be discussed and contrasted with the characterization results throughout the manuscript.

## 2. Materials and Methods 

Nanostructured magnetite iron oxides were synthesized by the coprecipitation of the corresponding iron hydroxide species formed in aqueous NH_4_OH medium, starting from the mixture of FeCl_2_·4H_2_O and FeCl_3_·6H_2_O reagents dissolved in deionized water, according to the following reaction:

FeCl_3_ + FeCl_2_ + 8 NH_4_OH → Fe_3_O_4_ + 8 NH_4_Cl + 4 H_2_O (1)

Magnetite-based solids differing in their specific surface areas were obtained using a nominal Fe^3+^/Fe^2+^ molar ratio of 2 or 4, and different coprecipitation temperatures (within the 80–110 °C range) in an inert atmosphere (N_2_) under reflux conditions. NH_4_OH was added at the corresponding synthesis temperature to get the basic medium for the iron hydroxides coprecipitation. In all cases the reflux temperature was maintained for 30 min. The precipitates were filtered, washed with deionized water, and dried at 100 °C/12 h.

(Ag)Pd-Fe_3_O_4_ nanocomposite materials were prepared by an impregnation procedure of Pd or Ag and Pd cations over the magnetite solid obtained by reflux at 80 °C with a nominal Fe^3+^/Fe^2+^ molar ratio of 4. The impregnation was carried out using the proper amount of the corresponding metal nitrate salt/s to get a metal load of 1, 2, or 3 wt.% Pd or 3 wt.% Ag-Pd. In the latter case, no sequential method was followed but both metals were simultaneously incorporated, employing an Ag:Pd molar ratio of 2:8. The impregnation procedure consisted of dissolving the Pd(NO_3_)_2_ and AgNO_3_ amounts needed in acetone and adding the magnetite solid into the acetone metal solution which was kept under stirring for 10 min, followed by an ultrasonication treatment for 30 min. This two-step cycle was repeated 4 times for every sample in order to favor a homogeneous dispersion of the noble metal/s over the magnetite. After the last cycle, the mixture was stirred at room temperature until the acetone was completely evaporated. The magnetite solid containing Pd or Ag/Pd was dried at 100 °C/12 h, and afterwards treated at 200 °C for 1 h, under a reducing gas stream consisting of an 80 mL min^−1^ total flow of H_2_/N_2_ (50/50), in order to selectively reduce the noble metal cations to their metallic state, avoiding the reduction of iron from the magnetite which starts over 350 °C.

XRD patterns were collected using an X’Pert PRO diffractometer with θ–2θ configuration, from PANalytical (Almelo, The Netherlands), equipped with an X’Celerator fast detector, operating at 45 kV and 40 mA and using a nickel-filtered Cu Kα radiation source which produces a nearly monochromatic X-ray beam (λ = 1.5406 nm). Diffractograms were obtained within the 4–90° 2θ range employing 20 s cumulative time.

The specific surface areas were determined by the Brunauer–Emmett–Teller (BET) method from N_2_ adsorption isotherms at 77 K measured in a Micromeritics ASAP2020 instrument (Norcross, GA, USA). A desorption treatment at 150 °C (10 °C min^−1^ heating rate) for 5 h, at high vacuum range conditions, was conducted for every sample just prior to the BET analysis.

The scanning electron microscopy analyses were performed employing a field emission scanning electron microscope (FESEM; model GeminiSEM 500, ZEISS, Oberkochen, Germany). Two different electron detection modes were used for FESEM imaging: (i) a secondary electron In-Lens detector, located inside the electron column, which works with low-energy secondary electrons and provides images with high resolution, and (ii) a energy selective backscattered (EsB) in-lens detector, independent of the secondary in-lens detector, which provides a pure backscattered signal with no secondary electron contamination and very low acceleration potential, providing a higher Z-contrast image than any other backscattered detector. Elemental chemical microanalyses in selected areas from the scanning electron micrographs were carried out with an X-ray energy-dispersive detector (EDS) from Oxford Instruments (Abingdon, UK), coupled with the FESEM microscope (ZEISS, Oberkochen, Germany).

A 200 kV analytical electron microscope equipped with a field emission electron gun (FEG; model JEM-2100F, JEOL, Tokio, Japan) allowing ultrahigh resolution in scanning transmission mode (STEM) through the sub-nanometric highly stable and bright electron probe (0.2 nm) provided by the FEG, was employed for the high-angle annular dark-field (HAADF) imaging in STEM mode. A high-resolution CCD (charge-coupled device) camera (2048 × 2048 pixels; model SC200, GATAN, Pleasanton, CA, USA), was used for image acquisition controlled by Digital Micrograph software. Image processing was performed by ImageJ free-software (Windows 10, 64-bit, version) [32]. The JEOL JEM-2100F microscope was equipped with a detector for X-ray energy-dispersive spectroscopy (EDS; model X-Max 80, Oxford Instruments, Abingdon, UK). The EDS detector, with 127 eV resolution and 0.5–2.4 nm spot size range, was used for elemental chemical analysis of punctual sites and selected areas from the STEM imaging.

Selected samples were measured by confocal micro-Raman spectroscopy using a LabRam-IR HR-800 model instrument (HORIBA Jobin Yvon, Edison, NJ, USA) coupled to an optical microscope model Olympus BX41. Excitation was provided by a He-Ne laser operating at a wavelength of 632.8 nm through a 100× objective lens, and with the laser power set to 0.6 mW, below the limit reported to induce phase transformations in iron oxides [33,34,35,36]. The regular Raman spectra were collected within the 100–1670 cm^−1^ region with 1 cm^−1^ spectral resolution, using an integration time of 8–16 s and 8–16 accumulations. In these conditions the lateral (xy) spatial resolution on the sample was 1 μm. For a high-resolution spectral profile, the integration time and the number of accumulations were increased to 32 s and 64, respectively, within a measuring region reduced to 320–777 cm^−1^.

Thermogravimetric analyses (TGA) were carried out in an SDT Q 600 apparatus (TA Instruments, New Castle, DE, USA) under reducing (H_2_) or oxidizing (synthetic air) conditions. Prior to analysis, samples were pre-treated at 150 °C for 1 h under a He stream (30 mL min^−1^ flow) which was maintained thereafter during the cooling down to room temperature. Following this pre-treatment, and according to the desired reducing or oxidizing experiment, the gas was switched from He to H_2_ (5 mL min^−1^ flow) or synthetic air (24 mL min^−1^ flow), respectively. After the adequate stabilization time (ca. 30–40 min.), the temperature was raised, at a 5 °C min^−1^ rate, to 200 °C; then until 800 °C using a different heating rate of 10 °C min^−1^. The evolution of species (CO, CO_2_, H_2_O, O_2_, N_2_, and H_2_) in the gas phase produced during the thermogravimetric experiments was followed with a quadrupole mass spectrometer (Pfeiffer Vacuum Omnistar™ GSD 301, Asslar, Germany) coupled with the TGA equipment. For this purpose, the following mass fragments (m/z) were measured: 2 (H_2_), 28 and 16 (CO), 44 (CO_2_), 18 and 17 (H_2_O), 32 and 16 (O_2_), 28 and 14 (N_2_).

The heterogeneous catalysis experiments for partial oxidation of methane were carried out in a fixed-bed quartz tubular flow reactor at atmospheric pressure, within the 100–250 °C temperature range, and using 100 mg of catalyst diluted with SiC (mg catalyst/SiC = ½ volume ratio). The feed consisted of a molar ratio CH_4_/O_2_/N_2_ = 32/4/64, for a total flow of 50 mL min^−1^. The separation and analysis of reactants and products were carried out online using a gas chromatograph equipped with two different chromatographic columns (3 m length molecular sieve 5 Å column and 30 m length RT-U-bond 0.53 mm i.d. column) and a thermal conductivity detector (TCD).

Blank reaction experiments up to 300 °C with no catalyst in the reactor tube showed no conversion at all. In all cases, carbon balances were 100 ± 4%, irrespective of the catalyst and mass used. Each data point for the assessment of catalytic performance was determined by averaging three different analyses of reactants and products. The minor differences observed among the repetitive analyses confirmed the steady-state conditions.

## 3. Results

### 3.1. Development of Nanostructured Fe_3_O_4_ Materials

**Figure 1:**
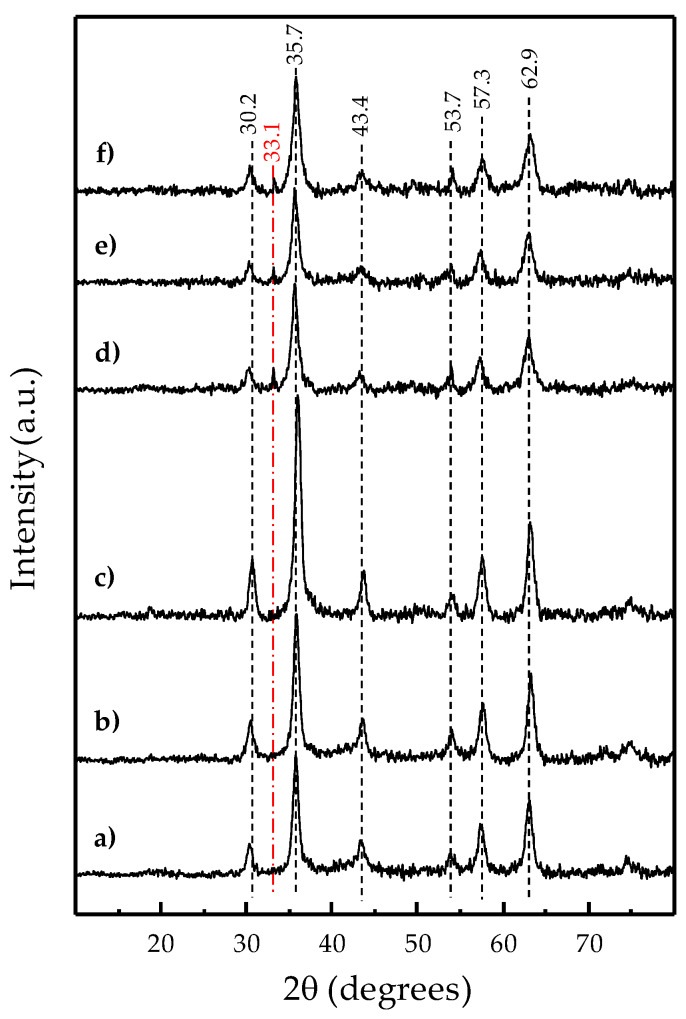
XRD patterns of magnetite solids prepared by coprecipitation employing a Fe^3+^/Fe^2+^ aqueous molar ratio of 2 (**a–c**) or 4 (**d–f**), at different synthesis temperatures: 110 °C (**a,d**), 95 °C (**b,e**), or 80 °C (**c,f**). Symbols: Fe_3_O_4_ (- - -), α-Fe_2_O_3_ (‒ · ‒ · ‒).

From the XRD patterns of the solids obtained by coprecipitation of the corresponding iron hydroxides during the reflux syntheses at different temperatures and employing aqueous mixtures of Fe^2+^ and Fe^3+^ chloride salts with Fe^3+^/Fe^2+^ molar ratio 2 or 4 (Figure 1), the presence of magnetite structure as the main phase formed in all cases can be confirmed. Thus, diffraction peaks at 2θ = 30.2, 35.7, 43.4, 53.7, 57.3, and 62.9° are present, with a similar intensity ratio among all the samples, according to the standard magnetite (Fe_3_O_4_) powder diffraction patterns previously reported [37,38,39]. 

It is important to point out that similar 2θ positions are found in powder XRD patterns of cubic maghemite (γ-Fe_2_O_3_) which moreover presents a medium intensity 2θ peak at 32.17° among others of lower intensity such as the ones at 15.0 and 23.8° [37,38,39,40]. The absence of those peaks confirms the assignment of magnetite as the major phase present in every iron oxide synthesized (Figure 1). Without limiting the foregoing, the XRD patterns of the solids prepared with a nominal Fe^3+^/Fe^2+^ molar ratio of 4 show an additional low-intensity peak at ca. 33.1°, which is not observed for those obtained from a nominal Fe^3+^/Fe^2+^ molar ratio of 2. The 2θ peak at 33.1° can be assigned to the presence of hematite (α-Fe_2_O_3_) as a minority phase since it appears as the highest relative intensity diffraction for the main rhombohedral hematite ICSD (Inorganic Crystal Structure Database) references (01-079-0007, 00-024-0072, 01-072-0469, 01-085-0599). Indeed, the hematite content was found below 10% from a semiquantitative analysis of the XRD data by X’Pert Highscore Plus software.

Despite this minor structural difference dependent on the nominal molar ratio used, a significant variation was found in the specific surface areas of the prepared iron oxides (Table 1) which additionally showed a dependence with the reflux temperature employed (varied from 80 to 110 °C). Thus, the BET surface area obtained for the Fe_3_O_4_ precipitates from the synthesis employing a nominal Fe^3+^/Fe^2+^ molar ratio of 4 was higher than those from a ratio of 2. For both ratios, the lower reflux temperature induced a higher specific surface area (Table 1).

Therefore, the highest BET area (138 m^2^ g^−1^) was achieved for the magnetite solid prepared at 80 °C employing a nominal Fe^3+^/Fe^2+^ molar ratio of 4, which was selected as the support for the following incorporation of Pd or Ag and Pd in order to prepare the target nanocomposite materials that would be catalytically tested for methane partial oxidation.

**Figure 2:**
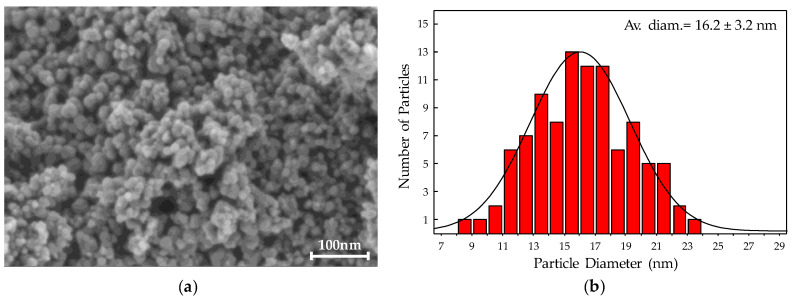
(**a**) Field emission scanning electron microscopy (FESEM) micrograph of magnetite solid precipitated from a reflux synthesis at 80 °C using a nominal Fe^3+^/Fe^2+^ molar ratio of 4. Image conditions: in-lens mode, work distance 2.7 mm, energy selective backscattered (EsB) grid of 300 V and electron high tension (EHT) of 1 kV. (**b**) Statistic distribution of particle diameter from several FESEM micrographs of the magnetite solid shown in (a).

From the analysis by field emission scanning electron microscopy (FESEM), a nanometric spherical morphology was found for the crystalline Fe_3_O_4_ particles prepared. An example is displayed in Figure 2 for the highest surface magnetite substrate selected to incorporate the noble metals in the next step. The elemental chemical microanalyses by X-ray energy dispersive spectroscopy (EDS), from a number of FESEM images showing similar homogeneous distribution of nanosized spherical particles, including those in Figure 2, confirmed an averaged Fe:O stoichiometry of 3.0 (±0.3):4.1 (±0.3) consistent with the magnetite Fe_3_O_4_ structure assigned from the powder XRD results (Figure 1). A representative statistic study of the crystallite size distribution from the FESEM micrographs of the highest surface magnetite, whose resulting histogram is displayed in Figure 2, illustrates an average Fe_3_O_4_ particle diameter of 16.2 ± 3.2 nm.

### 3.2. Development of Nanocomposite (Ag)Pd-Fe_3_O_4_ Materials

**Figure 3:**
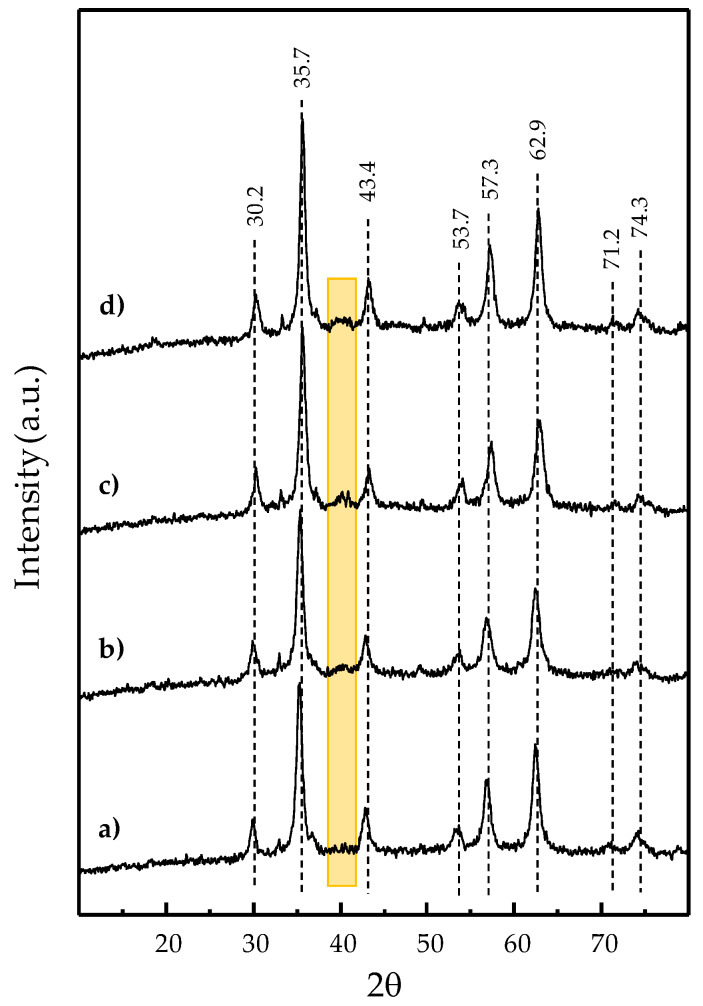
XRD patterns of nanocomposite catalysts based on magnetite (from 85 °C synthesis using Fe^3+^/Fe^2+^ molar ratio of 4) doped with Pd or Pd_-_Ag: 1% Pd-Fe_3_O_4_ (**a**), 2% Pd-Fe_3_O_4_ (**b**), 3% Pd-Fe_3_O_4_ (**c**), 3% AgPd-Fe_3_O_4_ (**d**). Symbols: (*- - -*) Fe_3_O_4_ (ICSD: 01-075-1609); the highlighted strip defines the region where the highest intensity diffractions for pure Pd, pure Ag, and AgPd alloys are found [41,42,43,44].

The incorporation of 1, 2, and 3 wt.% Pd, or 3 wt.% Ag-Pd (molar Ag:Pd ratio of 2:8) to the selected magnetite substrate by impregnation and subsequent reduction treatment (250 °C/1 h under 50% H_2_ in N_2_ stream), gave rise to the nanostructured (Ag)Pd-Fe_3_O_4_ composite materials that were tested as catalysts for methane partial oxidation. The XRD patterns of these nanocomposites are shown in Figure 3. All display the 2θ peaks common to those found for the (Ag)Pd-free iron oxide substrate (Figure 1f); i.e., the peaks’ set of 30.2, 35.7, 43.4, 53.7, 57.3, and 62.9° assigned to magnetite [37,38,39], along with the one at 33.1° from traces of hematite (ICSD: 01-079-0007). However, low-intensity 2θ peaks at 71.2 and 74.3° clearly appear for all the (Ag)Pd-containing magnetite samples (Figure 3). These two peaks can also be glimpsed, to a greater or lesser extent, in some XRD patterns of the pristine iron oxides synthesized from Figure 1, especially in those obtained from Fe^3+^/Fe^2+^ = 2 synthesis. A thorough analysis, comparing some representative references for indexed XRD patterns of Fe_3_O_4_ structures, allows us to identify that the set of the given main six peaks assigned above to Fe_3_O_4_ structure is common for both the cubic (ICSD: 01-076-0957, 01-075-0449) and the orthorhombic (ICSD: 01-075-1609) crystal systems of magnetite. Nevertheless, the low-intensity 2θ peaks at 71.2 and 74.3° appear specifically characteristic of orthorhombic magnetite.

Furthermore, semiquantitative analysis of the XRD data by X’Pert Highscore Plus software confirmed the major presence of an orthorhombic Fe_3_O_4_ structure in all (Ag)Pd-Fe_3_O_4_ catalysts from Figure 3. Only for 1% Pd-Fe_3_O_4_ and 3% AgPd-Fe_3_O_4_ samples did the semiquantitative phase analysis provide a minor coexistence with the cubic Fe_3_O_4_ structure (ca. 10% and 20%, respectively). 

On the other hand, an additional low-intensity peak at 2θ = 40.1° can be observed for the 3% Pd-Fe_3_O_4_ catalyst (Figure 3c), which corresponds to the (111) plane of metal Pd [41,42,44]. This low-intensity peak turns into a small broadband within the 2θ range from 38.7 to 41.6° for the bimetallic 3% AgPd-Fe_3_O_4_ sample. This band, along with the glimpsed wide bump centered at 45.5°, is in full agreement with previously reported XRD patterns of bimetallic AgPd alloys presenting fcc crystalline structure [41,42,43,44], especially with those containing Ag:Pd molar ratio of 2:8 [42] or between 3:7 and 1:9 [41]. The main XRD diffraction of metal Pd is barely observed for the 2% Pd-Fe_3_O_4_ sample and the region becomes totally flat for the 1% Pd-Fe_3_O_4_, which is coherent to the quantitative detection limit (<2%) associated to the XRD technique for multiphase mixtures [45].

**Figure 4:**
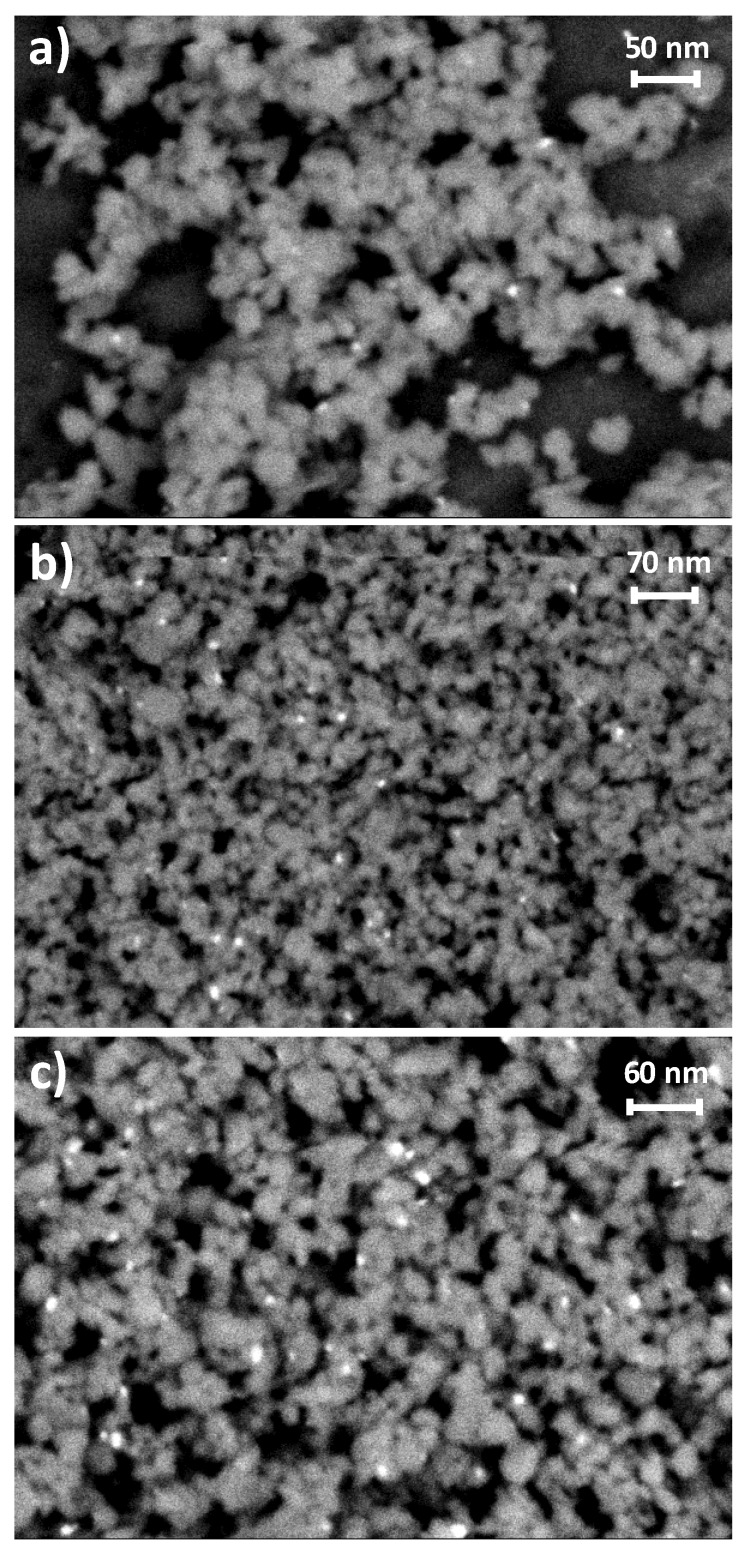
Z-contrast micrographs obtained by high-resolution field emission scanning electron microscopy (HRFESEM) using an energy selective backscattered (EsB) electron in-lens detector for the Fe_3_O_4_-based catalysts doped with 1% Pd (**a**), 2% Pd (**b**), and 3% Pd (**c**).

The monometallic Pd-Fe_3_O_4_ samples were investigated by field emission scanning electron microscopy (FESEM) using an energy selective backscattered (EsB) electron in-lens detector which can select the electrons according to their energy. Thus, the EsB detector is very sensitive to variations in the atomic number (Z) of atoms, allowing Z-contrast images (i.e., brighter image appearance for atoms with a higher Z), since the higher the atomic number the more electrons are scattered due to greater electrostatic interactions between the nucleus and the microscope electron beam at high angles. Indeed, Figure 4 shows Z-contrast micrographs for 1% Pd-, 2% Pd-, and 3% Pd-Fe_3_O_4_ samples, all with glaring bright spots consistent with metal Pd clusters, over a matt grey matrix assigned to Fe_3_O_4_. Congruently, a higher population density of these metal clusters is displayed as the nominal Pd load increases in the magnetite (Figure 4).

The average diameter of the metal Pd clusters was statistically determined for each Pd-Fe_3_O_4_ specimen. By direct measurement using ImageJ software [32], over more than 150 shiny clusters from several micrographs were acquired by EsB mode. Thus, the results in Table 2 show an average diameter of 3.3 ± 1.2 nm for the Pd clusters in 1% Pd-Fe_3_O_4_ catalyst, which remains practically unchanged for 2% Pd-Fe_3_O_4_, while it slightly increases to 4.4 ± 1.6 nm for the highest Pd load (i.e., 3% Pd-Fe_3_O_4_). It should be mentioned that the EsB detector employed gives a higher Z-contrast than any other backscattered detector, enabling differentiation between elements that are only distinguished by a few atoms.

**Figure 5:**
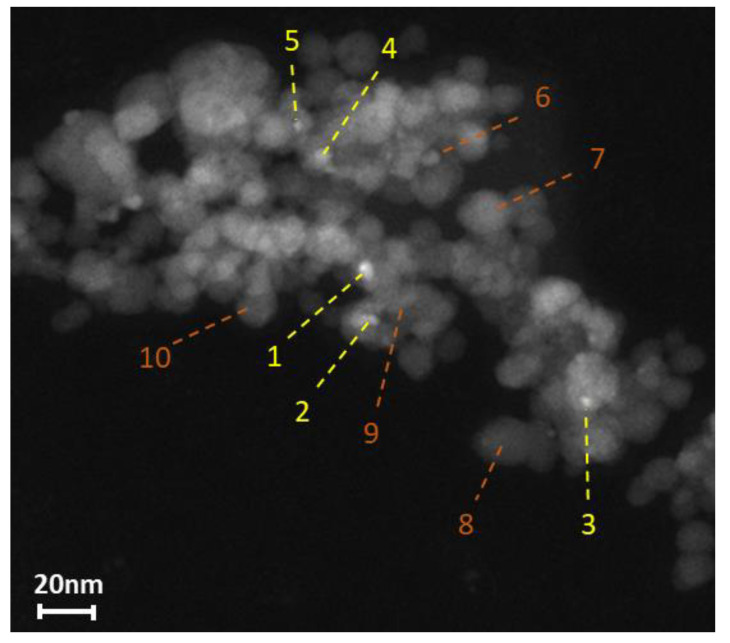
HAADF-STEM micrograph of 3% AgPd-Fe_3_O_4_ sample, with SA-EDS analysis spots marked and numbered: chemical analyses in brighter particles (1–5 spots) showed AgPd wt.% >12, with an averaged Ag:Pd atomic ratio of 0.2:0.8 (0.1 SD), while in 6–10 spots AgPd wt.% <0.9.

For the bimetallic 3% AgPd-Fe_3_O_4_ sample, the Z-contrast microscopy studies were carried out differently, employing high-angle annular dark-field (HAADF) imaging in scanning transmission electron mode (STEM), using an analytical electron microscope equipped with a field emission electron gun (FEG). In a similar way to the EsB detector from the high-resolution field emission scanning electron microscopy (HRFESEM) instrument, the HAADF in STEM senses a greater signal from atoms with a higher atomic number (i.e., causing the metal atoms to shine brighter in the resulting image). Indeed, HAADF-STEM micrographs of 3% AgPd-Fe_3_O_4_, as the example displayed in Figure 5, show dull grey spheres within the diameter range of 11–20 nm, accordingly with Fe_3_O_4_ nanospheres, underneath some brighter and smaller metal particles with an average diameter of 4.3 nm (Table 2). The chemical analysis pointed to these metal particles (Figure 5) using selected area X-ray energy-dispersive spectroscopy (SA-EDS) with 0.5–2.4 nm spot size range (Table 2) and confirmed the coexistence of Ag and Pd with an averaged Ag:Pd stoichiometry of 0.2:0.8 (±0.1), consistent with the Ag_0.2_Pd_0.8_ alloy structure observed by XRD (Figure 3).

### 3.3. Catalytic Tests for Methane Partial Oxidation

**Figure 6:**
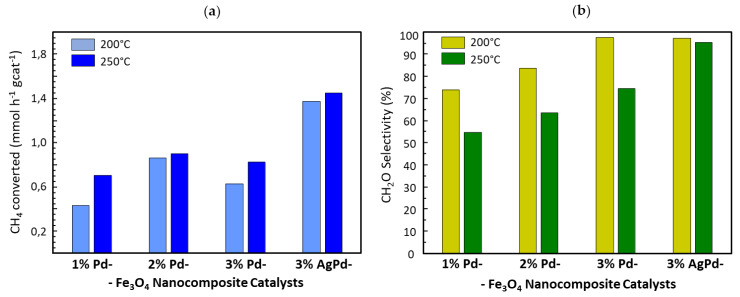
Catalytic activity (**a**) and selectivity to formaldehyde (**b**) for methane partial oxidation over the (Ag)Pd-Fe_3_O_4_ nanocomposite catalysts at 200 °C (light color bars) and 250 °C (dark color bars). Reaction conditions: 100 mg catalyst mass, CH_4_/O_2_/He molar ratio of 32/4.3/63.7, and contact time (W/F, at standard conditions) of 2.6 g_cat._ h mol_CH4_^−1^.

The catalytic properties of the developed nanocomposite (Ag)Pd-Fe_3_O_4_ materials were tested in gas phase heterogeneous catalysis for the partial oxidation of methane at moderate reaction conditions (i.e., ambient pressure and temperatures up to 250 °C). Interestingly, using these mild conditions, methane gets activated although with low reaction rates. The catalytic results, summarized in Figure 6, show methane conversion at 200 °C for all the catalysts tested, leading to the formation of formaldehyde as the main reaction product (>74%), with CO_2_ as the only secondary product detected. In general, the bimetallic 3% AgPd-Fe_3_O_4_ catalyst shows a catalytic activity significantly higher than the monometallic Pd-Fe_3_O_4_ catalysts; the latter ones presenting a maximum activity for the 2% Pd-Fe_3_O_4_ sample (Figure 6a). Therefore, no linear correlation is observed between the catalytic activity and the formal content of Pd which could be initially considered as the major source of methane activation at such a low temperature, according to the literature [30,31]. On the other hand, the selectivity to formaldehyde rises with the increase in the Pd load, showing a maximum above 97% for both the monometallic 3% Pd-Fe_3_O_4_ catalyst and the bimetallic 3% AgPd-Fe_3_O_4_ one at 200 °C (Figure 6b). At 250 °C the methane conversion slightly increases, while the selectivity to the partial oxidation product proportionally drops for all the monometallic Pd-Fe_3_O_4_ catalysts, with no absolute inverse correlation between conversion and selectivity observed. On the contrary, the high selectivity to formaldehyde hardly changes for the bimetallic 3% AgPd-Fe_3_O_4_ catalyst from 200 to 250 °C at which a formaldehyde productivity of 43 g_CH2O_ kg_cat_^−1^ h^−1^ is reached with a turnover frequency (TOF) of 24.7 h^−1^ (reaction conditions are shown in the footnote of Figure 6).

The pristine metal-free iron oxide substrate was comparatively tested for the partial methane oxidation upon identical reaction conditions used in the (Ag)Pd-Fe_3_O_4_ nanocomposite catalysts, detecting no methane conversion, likewise the blank results obtained no catalyst. Moreover, the catalytic results were confirmed by testing twice, under equivalent reaction conditions, all the nanocomposite catalysts displayed in Figure 6. The catalysts used in the first test were employed in a second catalytic cycle showing no apparent change in their performance (i.e., similar catalytic activity and selectivity).

## 4. Discussion

According to the results presented, the experimental research of this work consisted of three basic stages: (i) the development of a high surface nanostructured Fe_3_O_4_ substrate; (ii) its coating with noble metal nanoparticles to form mono- or bi-metallic (Ag)Pd-Fe_3_O_4_ nanocomposites; (iii) to assess the potential of the noble-metal/iron-oxide nanocomposites developed as catalysts for gas-phase partial oxidation of methane at ambient pressure and mild temperatures. 

For the first stage, a reflux-assisted coprecipitation was the chosen method to carry out the preparation of high surface optimized magnetite. In this sense, a systematic study is presented here on the influence of both the reflux temperature (80–110 °C range) and the aqueous Fe^3+^/Fe^2+^ molar ratio (2 or 4) in the specific surface area of the resulting magnetite precipitate. The data analysis confirmed a higher Fe_3_O_4_ surface area is strongly favored for Fe^3+^/Fe^2+^ molar ratio of 4, rather than 2, in the aqueous synthesis solution (Table 2). Regardless, the magnetite surface area was also observed to increase as the reflux temperature was lowered within the 110–80 °C range, for both nominal ratios assayed (Table 2). At this point, it should be mentioned that an aqueous Fe^3+^/Fe^2+^ molar ratio of 2, matching the Fe_3_O_4_ stoichiometry, has been traditionally employed in the coprecipitation-based methods reported for magnetite preparation [37,46,47,48,49]. In fact, no peer-reviewed publication could be found, with which to compare our results, that claimed the use of Fe^3+^/Fe^2+^ = 4 for Fe_3_O_4_ synthesis. Nevertheless, the gain in the specific surface area achieved by the Fe_3_O_4_-based solid prepared by the higher nominal Fe^3+^/Fe^2+^ ratio in synthesis (Table 2) is in good agreement with a size decrease reported to be observed for Fe_3_O_4_ particles in suspension while increasing Fe^3+^/Fe^2^ ratios [49]. However, along with this statement, in the first known work employing a coprecipitation method for magnetite preparation, no numeric values for Fe^3+^/Fe^2+^ ratios larger than 2 are overtly mentioned.

Notwithstanding the excess of Fe^3+^ present in the synthesis media when using an Fe^3+^/Fe^2+^ ratio of 4, compared with the Fe_3_O_4_ stoichiometry, the elemental and structural analyses of the higher surface area precipitates are consistent with a major presence of the mixed iron oxide bulk phase (just like the solids from stoichiometric Fe^3+^/Fe^2^+ ratio synthesis). On the one hand, an averaged Fe:O stoichiometry of 3.0:4.1 (± 0.3), especially consistent with magnetite, was experimentally determined by EDS microanalyses on nanospheres, with 16.2 nm average diameter (Figure 2), to make up the ca. 140 m^2^·g^−1^ solid (Table 2). On the other hand, the 2θ peaks at 30.2, 35.7, 43.4, 53.7, 57.3, and 62.9° present in the corresponding XRD patterns (Figure 1d–f) are consistent with the (2 2 0), (3 1 1), (4 0 0), (4 2 2), (5 1 1), and (4 4 0) planes of standard magnetite structure (Joint Committee on Powder Diffraction Standards, JCPDS, file No.: 19-0629), respectively. These diffractions are also common to the γ-Fe_2_O_3_ structure (JCPDS file No.: 04-0755) whose assignment as major bulk phase was discarded by the absence of its additionally associated peaks from (210), (300), and (320) planes of crystalline maghemite (γ-Fe_2_O_3_) [39]. Nevertheless, a minor presence of maghemite cannot be completely ruled out. In fact, many studies claim that it is not straightforward to observe pure magnetite or maghemite, but an intermediate defective structure, Fe_3-x_O_4_, between both [50,51,52,53]. This has been especially proven for particles within the nanometric scale [52,53,54,55,56], for which a magnetite core with a maghemite shell structure is widely reported [48,54,55,56]. For the most oxidized iron oxide layer, in the cited core-shell structures, a varying thickness has been found depending on different factors such as the accurate nanoparticle size [54] and/or the reactants used and other preparation conditions [48,57].

**Figure 7:**
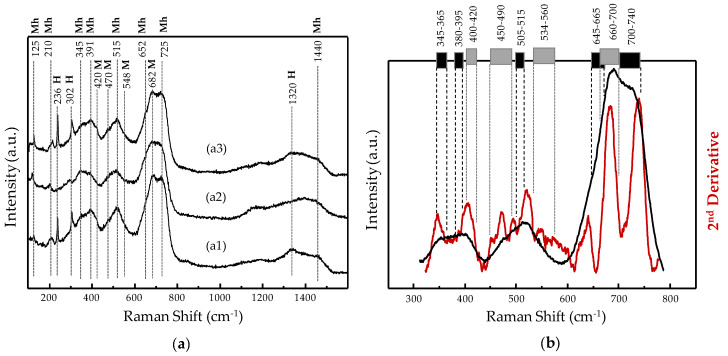
(**a**) Raman spectra of pristine Fe_3_O_4_-based substrate (a1), 3% Pd-Fe_3_O_4_ (a2), and 3% AgPd-Fe_3_O_4_ (a3) nanocomposite catalysts, collected at 0.6 mW. (**b**) High-resolution Raman spectrum and its second derivative by the Savitzky–Golay algorithm, using 81 convolution points of the Fe_3_O_4_-based substrate submitted to identical reduction treatment (250 °C/2 h in H_2_) as the noble metal-containing catalysts. Acronyms from (a): *Mh* (maghemite), *H* (hematite), and *M* (magnetite) label the dotted lines according to the assignment ranges found in the literature (references at the end of the footnote). Symbols from (b): numerical ranges together with the black and grey labels define the regions where maxima have been reported in the literature for maghemite [33,61,62,63,64,65] and magnetite [33,34,58,59,60,61,62,63].

Due to the inherent short-range periodic order that maghemite would exhibit in such a scenario, our XRD data would not be useful to directly reveal or deny its minor coexistence together with the magnetite structure. In this respect, Raman spectroscopy was complementarily employed to analyze some representative samples whose spectra are displayed in Figure 7. Thus, the Raman spectrum of the highest surface Fe_3_O_4_-based substrate used to incorporate the noble metals (Figure 7, spectrum a1) does not present a typical pure magnetite profile which is considered to be characterized by a single narrow and intense band reported to be centered within the 660–700 cm^−1^ range, together with a weak one between 534 and 560 cm^−1^ [33,34,58,59,60,61,62,63]. Instead, the Raman spectrum for the metal-free Fe_3_O_4_-based substrate is mainly characterized by the features assigned to maghemite (γ-Fe_2_O_3_) in the scientific literature [33,61,62,63,64,65]; i.e., a broad and intense asymmetric band from ca. 600 to 800 cm^−1^, assigned to *A*_1g_ vibrational mode, and two more bands also asymmetric but weaker: one with two maxima reported to be centered at 345–365 cm^−1^ (*E*_g_ mode) and 380–395 cm^−1^, and another one centered at 505–515 cm^−1^ (*T*_1g_ mode) [62,65,66]. In addition, two narrow peaks with relatively low intensity appear at 236 and 302 cm^−1^, matching with the strongest vibrational bands (*A_1g_* and *E_g_* modes, respectively) related to hematite [35,62,63,64,65], along with the broadband centered at 1320 cm^−1^ [35,67,68]. In this sense, it must be said that Raman spectroscopy is extremely sensitive to the presence of hematite. Even traces of hematite impurities contained within other major iron oxides (as it is known in our case from XRD results) are able to provide narrow peaks with spurious intensity due to the large scattering power for Raman radiation inherent to hematite [60], inducing wrong conclusions about its relative content unless complementary characterization techniques are also employed.

Regarding maghemite, its strongest band is usually described as consisting of two components whose maxima are reported within 645–665 cm^−1^ and 700–740 cm^−1^ ranges [48]. Additionally, a magnon band centered at ca. 1440 cm^−1^ and two low-intensity peaks at 125 and 210 cm^−1^ are also typical of maghemite structure [66,69]. Unfortunately, the characteristic Raman features linked to magnetite (at ca. 547 and 680 cm^−1^) overlap with the three major bands cited for maghemite, which make difficult unambiguous statements about magnetite just based on the Raman spectrum when maghemite is present as well. A tool that might help to clarify this is the second derivative of the Raman spectrum. For this purpose, a high-resolution Raman spectrum within the region of interest was collected for the Fe_3_O_4_-based substrate, this time after being submitted to the same reduction treatment (250 °C/2 h in H_2_) as the noble metal-containing samples. The spectrum obtained and its second derivative are displayed in Figure 7b. Regardless of the higher resolution, the Raman spectrum is very similar to that obtained from the pristine substrate within the same region (Figure 7a1) which leads to identical conclusions. Nevertheless, the second derivative shows a resolved accurate position for the maxima associated to the vibrational Raman modes among which it is possible to distinguish a maximum within the 534–560 cm^−1^ region (Figure 7b), where no maximum but the one from a weak vibrational mode of magnetite is reported [63,66].

Therefore, Raman results represented by the spectra in Figure 7, provide unambiguous evidence for the presence of maghemite. Unfortunately, further conclusions about either the structural configuration or the phases distribution of the given iron oxide mixture cannot be done from Raman data, due to the intimate overlapping between the main Raman band of magnetite and one of the strongest bands of maghemite (Figure 7). Furthermore, the ultrathin maghemite layer derived from a minor magnetite surface oxidation cannot be completely ruled out, although laser power was strictly kept far below the limits reported for the laser-induced magnetite oxidation [33,34]. In this respect, transformation into maghemite at temperature values as low as 109 °C has been reported for magnetite nanoparticles presenting 13–16 nm diameters (i.e., within the range measured for our Fe_3_O_4_ substrate) (Figure 2).

Moving forward to the discussion from the second experimental stage, the incorporation of noble metal nanoparticles to the magnetite-based substrate (the one with the highest surface area), followed by the given selective reduction treatment, leads to the corresponding mono- or bi-metallic (Ag)Pd-Fe_3_O_4_ nanocomposites (Table 2). Raman spectra of the representative catalysts 3% Pd-Fe_3_O_4_ and 3% AgPd-Fe_3_O_4_ are displayed as spectrum a2 and a3, respectively, in Figure 7a. These spectra show meaningless differences for the mixed iron oxide vibrational features compared with the pristine metal-free substrate, apart from the greater or lesser intensity of the narrow peaks at 236 and 302 cm^−1^ from the hematite impurity traces. Therefore, identical conclusions as those discussed above apply here.

With respect to the crystalline phases from the XRD results, new features appear for metal Pd or AgPd alloy structures, along with those remaining from the Fe_3_O_4_ substrate (Figure 3). On the other hand, no intensity is detected for the main diffraction of either metal Fe [70] or PdO [71,72,73] at 44.7 and 33.9° 2θ, respectively, which is consistent with the selective and complete noble metal reduction. The average diameter for the metal clusters was found to increase from ca. 3.3–3.4 nm for 1% Pd- and 2% Pd-Fe_3_O_4_ samples, to ca. 4.3–4.4 nm for the 3% Pd- and 3% AgPd-Fe_3_O_4_ ones. In the specific case of the bimetallic nanocomposite sample, the stoichiometry determined in the solid by structural and elemental analysis (i.e., by XRD (Figure 3) and by SA-EDS from HAADF-STEM imaging (Figure 5 and Table 2), respectively) was consistent with the nominal Ag:Pd stoichiometry used (0.2:0.8) during the impregnation treatment. Although we did not employ surfactant or any other sophisticated method to control the homogeneous size of the noble metal particles during the incorporation treatment, the standard deviation determined for the size distribution of (Ag)Pd nanoparticles on the magnetite-based substrate was acceptable (ca. 33%) in both mono- and bi-metallic nanocomposite samples (Table 2).

Finally, with respect to the stage of catalytic reactivity evaluation, promising results were obtained for the gas-phase methane partial oxidation into formaldehyde (Figure 6), for which the catalytic system (Ag)Pd-Fe_3_O_4_ was never reported, to the best of our knowledge. The highest formaldehyde productivity was shown by the 3% AgPd-Fe_3_O_4_ catalyst, yielding ca. 45 g_CH2O_ kg_cat_^−1^ h^−1^ with a turnover frequency (TOF) of 24.7 h^−1^ (calculated for a 26% Pd dispersion) at 250 °C, far below the reaction temperatures reported so far [8,12]. In this sense, oxidation of methane has been hardly reported to occur below 600 °C [8,10,12]. Thus, the catalytic data provided by most of the studies on methane selective oxidation to formaldehyde correspond to reaction temperatures within the 600–800 °C range [8,12]. Ironically, formaldehyde decomposing into methanol and carbon monoxide starts at temperatures above 150 °C, although the kinetics for the uncatalyzed reaction is slow below 300 °C [74]. As a matter of fact, 450 °C appears as the lowest temperature reported so far, providing heterogeneous catalysis data of formaldehyde yielded by methane oxidation using molecular oxygen [75]. Nonetheless, the use of N_2_O as a more reactive oxygen donor has been reported to run methane partial oxidation at 350 °C on several Fe-doped zeolites, although no selectivity to formaldehyde above 8% is claimed in that work [9]. Instead, a dispersion of partial oxidation products (CH_3_OH, CH_2_O, and C_2_H_4_) with low total selectivity was obtained, close to 40% in the best case [9]. On the contrary, selectivity to formaldehyde above 95% is achieved at 200–250 °C with the 3% AgPd-Fe_3_O_4_ catalyst developed in the current work (Figure 6). In addition to the major formation of formaldehyde, CO_2_ was the only byproduct obtained in our catalytic tests for methane partial oxidation. No trace of methanol or CO was detected as byproducts, suggesting no formaldehyde decomposition. Moreover, reported pure radical mechanisms for aldehyde formation via methanol intermediate and/or consecutive decomposition involving CO formation, before or along with CO_2_ production [8], can be discarded as plausible mechanisms being held on our (Ag)Pd-Fe_3_O_4_ catalysts. Instead, the so-called ion-radical mechanism, reported to involve electron transfers from the catalyst lattice oxygens, appears the most consistent with our catalytic results. Thus, this mechanism justifies the exclusive formation of formaldehyde and/or CO_2_, by single-electron transfer from O^–^ centers and/or two-electron transfer from O_2_^2–^ centers, respectively, both at the catalyst surface [8]. The ion-radical mechanism has been reported for catalysts based on metal oxides containing ambivalent metals that are able to easily change their oxidation states, such as MoO_3_ or V_2_O_5_. As a matter of fact, these two oxides supported on SiO_2_ are the catalytic systems grabbing a majority of studies concerning the oxidation of methane into formaldehyde [4,5,6,7]. Similarly, the cited ambivalence is also present in the mixed iron oxide from magnetite, the major component in our catalytic system. In fact, meaningful results in methane partial oxidation to formaldehyde have been reported for several catalysts containing iron oxide species [3,8,9,10,11,12,13,14]. The most encouraging finding to keep deploying resources in CH_4_ conversion technology is, indeed, nature’s iron-containing active center located in the methane monooxygenase enzyme able to selectively transform CH_4_ into methanol, formaldehyde, and formic acid at ambient conditions [76,77]. Moreover, along with Mo and V, Fe was in the six elements list whose SiO_2_-supported oxides were concluded to be the most suitable active phases for methane partial oxidation based on the screening of 43 elements [78]. However, no specific mention to the use of Fe_3_O_4_ in methane direct conversion reactions has been found in the literature, neither as an active phase nor support. With respect to the latter, in addition to SiO_2_, other high surface substrates such as Al_2_O_3_, TiO_2_, SnO_2_, and zeolites are among the most used for supporting the active phases for partial oxidation of methane.

**Figure 8:**
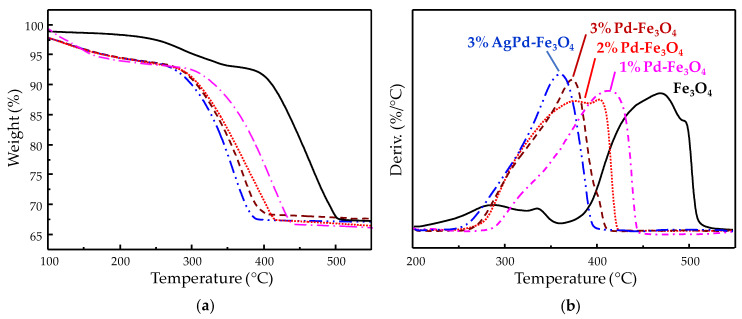
Thermogravimetry analysis curves collected in H_2_ for the different (Ag)Pd-Fe_3_O_4_ catalysts prepared and for the pristine metal-free Fe_3_O_4_ substrate (**a**), and their first derivative (**b**).

Noble metals such as Pd or Pt have also been briefly studied as active phases for methane activation, compared with transition metal oxides. Particularly, nanosized Pd has been reported to be highly reactive at temperatures as low as 200 °C, although toward complete methane oxidation [79]. Despite some interesting proposals for inhibiting the uncontrolled Pd activity leading to total combustion products [12,79], noble metals were practically left out in research on methane partial oxidation. Within this context, the nanocomposite material addressed in the current work, based on Fe_3_O_4_-supported (Ag)Pd, appears as a relatively known system, but a novel type of catalyst for the methane direct conversion into oxygenated partial oxidation products. The initial working hypothesis was to comparatively incorporate Ag with the purpose to downgrade the expected overreactive nanostructured Pd. In this regard, Ag was reported to selectively segregate at the surface of nanostructured Pd, suppressing the sites with the strongest oxidizing reactivity [30,31]. Indeed, the highest catalytic performance is achieved with the Fe_3_O_4_-based catalyst containing the bimetal AgPd alloy (Figure 6). Thus, the results settle that Ag improves selectivity toward the partial oxidation product at higher catalytic activity, which suggests confirmation of the working hypothesis about a softening effect on Pd reactivity. However, in general, the catalysts consisting of pure Pd supported over Fe_3_O_4_ have surprisingly shown high formaldehyde selectivity as well, rather than the total combustion products expected according to the published literature on Pd predisposition toward complete methane oxidation [79]. Even more, the selectivity to formaldehyde rises with increasing Pd load (from 1 to 3 wt.%). Regarding the assessment of catalytic activity from the pristine Fe_3_O_4_-based substrate itself, it could be dismissed by getting no conversion at all for the methane oxidation at identical reaction conditions used for the (Ag)Pd-containing catalysts. The latter statements might suggest a kind of synergy effect between Pd and the magnetite substrate. In this sense, by means of thermogravimetry analyses under reducing or oxidizing conditions, a relationship was found between the redox properties of the iron cations and the Pd content added to the mixed iron oxide. Thus, Figure 8b displays the derivative of thermogravimetry curves (DTG) collected under a H_2_ stream (i.e., reducing conditions), with the positive peak maximums in the graph representing the greatest rates for each weight loss step which correspond to each dehydration accompanying a reduction. For the metal-free mixed iron oxide substrate, two separated reduction stages can be distinguished, namely, a small peak at low temperatures and a large one at a higher temperature range, below and above 360 °C, respectively. The first region between 200 and 360 °C corresponds to the reduction of minority pure Fe (III) oxides to Fe_3_O_4_. This reduction is clearly comprised of at least two overlapped peaks with maxima at 284 °C and 334 °C which, according to the reported literature [80], should correspond to the reduction of maghemite (γ-Fe_2_O_3_) to Fe_3_O_4_ and hematite (α-Fe_2_O_3_) to Fe_3_O_4_, respectively. This assignment is also consistent with the hematite impurity observed by XRD (Figure 1) and the homogeneous minor presence of maghemite proved to coexist with magnetite by comparing Raman spectroscopy (Figure 7) and XRD results (Figure 1). According to the following stoichiometry: 3Fe_2_O_3_ + H_2_ → 2Fe_3_O_4_ + H_2_O; a Fe_2_O_3_ content (γ-Fe_2_O_3_ + α-Fe_2_O_3_) of ca. 2% was calculated from the weight loss within the 200–360 °C region during thermogravimetry (TG) analysis in H_2_ of the metal-free Fe_3_O_4_-based substrate (Figure 8a). On the other hand, the second region, from 360 °C to 550 °C, reflects major reduction consisting of a two-step Fe_3_O_4_ reduction sequence to Fe^0^ through wüstite (FeO) intermediate formation [81].

The Pd incorporation on Fe_3_O_4_ leads to nanocomposite materials presenting a reduction temperature lower than the pristine magnetite-based substrate, either for the one-step reduction from minority Fe_2_O_3_ phases to Fe_3_O_4_ or the two-step reduction from Fe_3_O_4_ to FeO and Fe^0^. Thus, the reduction temperature for the minor Fe_2_O_3_ phases moves bellow 200 °C, in agreement with previous publications employing Pd or other noble metals, such as Au, on Fe_2_O_3_ [80,82]. In Figure 8a, it can be observed that the corresponding weight loss for (Ag)Pd-Fe_3_O_4_ catalysts starts already at 100 °C, while the beginning of the equivalent loss for the pristine substrate delays until 200 °C.

The derivative TG curve, within the major reduction region, is displayed in Figure 8b, where the maximum of the major peaks correspond to the temperature for the maximum rate of Fe_3_O_4_ reduction which decreases following the next trend: Fe_3_O_4_ (468 °C) > 1% Pd-Fe_3_O_4_ (412 °C) > 2% Pd-Fe_3_O_4_ (376 °C) > 3% Pd-Fe_3_O_4_ (370 °C) > 3% AgPd-Fe_3_O_4_ (358 °C). According to this sequence, the Fe_3_O_4_ reducibility seems to be enhanced as the Pd load increases (from 1 to 3 wt.%) in the monometallic catalysts. Nevertheless, the tendency breaks for the 2.4 wt.% Pd load in the bimetallic 3% AgPd-Fe_3_O_4_ catalyst for which the doping with Ag forming the Ag_0.2_Pd_0.8_ alloy appears to favor the lowest temperature for Fe_3_O_4_ reduction at 358 °C (i.e., the highest reducibility).

**Figure 9:**
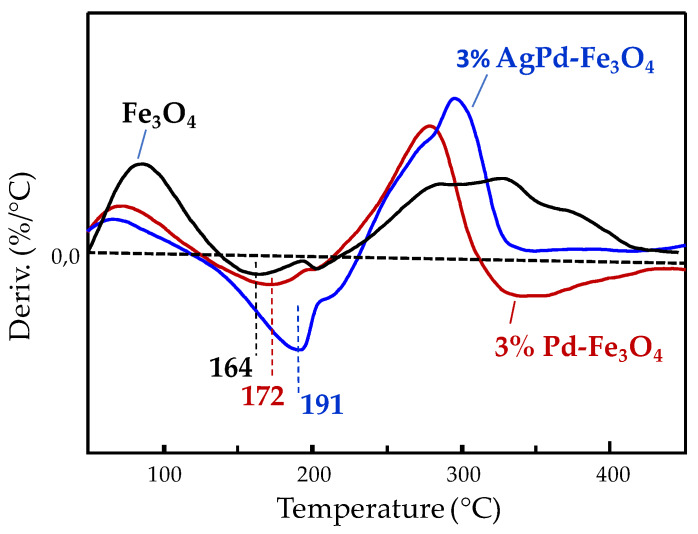
The first derivative of the thermogravimetry (TG) curves collected under synthetic air flow for the monometallic 3% Pd-Fe_3_O_4_ and the bimetallic 3% AgPd-Fe_3_O_4_ catalysts, as well as for the pristine metal-free Fe_3_O_4_-based substrate.

A thermogravimetry (TG) analysis under oxidizing conditions (dry air stream) was also performed in three representative samples, in order to distinguish whether the effect on the redox properties of iron species, observed by the drop in their reduction temperature, goes further than the spillover effect of atomic hydrogen produced by catalyzed H_2_ dissociative adsorption on Pd [83,84]. Thus, Figure 9 displays the first derivative of the TG curve collected under synthetic air flow for 3% Pd-Fe_3_O_4_ and 3% AgPd-Fe_3_O_4_ catalysts, and their pristine Fe_3_O_4_ substrate. The first negative peaks observed in Figure 9 within the 150–200 °C range correspond to the maximum rate of weight gain produced by oxygen insertion causing Fe^2+^ oxidation to Fe^3+^, for which the corresponding temperature, appearing at 164 °C in the metal-free Fe_3_O_4_ increases in the noble metal-containing catalysts, especially in the bimetallic 3% AgPd-Fe_3_O_4_ with the maximum at 191 °C. Therefore, the redox properties of iron species undergo generalized changes induced by the Ag and/or Pd nanostructures supported, whereby the Fe^3+^ species become easier to reduce (i.e., present higher oxidizing power), while the Fe^2+^ ones become harder to oxidize. The strong correlation, existing between the trend in the change of the mixed iron oxide redox properties and the increased productivity to formaldehyde, points to a significant metal–support interaction that seems to be responsible for the tendency observed in the catalytic behavior of the different nanocomposite catalysts.

## 5. Conclusions

From the systematic study on the synthesis of Fe_3_O_4_-based solid with an improved specific surface area, an enhancement from 80 to 140 m^2^ g^−1^ was achieved by doubling the nominal Fe^3+^/Fe^2+^ ratio from 2 to 4 and decreasing the synthesis temperature from 110 to 80 °C. (Ag)Pd-Fe_3_O_4_ nanocomposite materials were developed through solvent-assisted (Ag)Pd impregnation on the Fe_3_O_4_-based substrate with optimized surface area, followed by a selective reduction treatment. The resulting nanocomposites can be defined as Pd metal or AgPd alloy nanoparticles with ca. 3.3–4.4 nm diameter supported on Fe_3_O_4_-based nanoparticles of 16.2 nm average diameter. The coexistence of maghemite was proven by Raman, although in a minor amount (<3 wt.%) according to XRD and TG results. Further research must be done in order to establish the accurate structural configuration linking the major Fe_3_O_4_ and the minor γ-Fe_2_O_3_ phases within the nanostructures developed.

Catalytic activity for methane conversion appears in all (Ag)Pd-Fe_3_O_4_ nanocomposite catalysts tested, at temperatures as low as 200 °C. The reported propensity toward methane combustion by nanostructured Pd has shown to be suppressed within the Pd-Fe_3_O_4_ nanocomposite systems (1–3 wt.% Pd load), which present a major selectivity to formaldehyde. The higher Pd load, within the studied range, increases the catalyst selectivity to formaldehyde which reaches above 95% for the catalysts containing above 2 wt.% Pd. On the other hand, a correlation was also observed between the increased Pd load and a gradually enhanced reducibility of the Fe^3+^ species in magnetite, which confirms a significant metal–support interaction. This reducibility in the Fe_3_O_4_ phase improves even further with the Ag incorporation forming the Ag_0.2_Pd_0.8_ alloy. These results suggest that the iron oxide substrate is most likely involved in the catalytic partial oxidation of methane into formaldehyde. This assumption is also supported by the CO_2_ formation as the only secondary product which has been reported to occur by the ion-radical mechanism involving electron transfers from the catalyst lattice oxygens in catalysts containing oxides from ambivalent metals.

The nanocomposites have proven worthy for further research in gas-phase methane partial oxidation at relatively low reaction temperatures, compared with those reported until now using other catalysts. Forthcoming works will increase the low catalytic activity observed and shed more light on the mechanisms, active sites involved, and the role of the metal–support interaction.

## Figures and Tables

**Table 1:** Specific surface areas of magnetite solids precipitated at different reflux temperature and nominal Fe^3+^/Fe^2+^ molar ratio.

Fe^3+^/Fe^2+^	Temperature (°C)	S_BET_ (m^2^ g^−1^) ^1^
2	110	77.0
2	95	91.3
2	80	96.9
4	110	125.7
4	95	135.8
4	80	137.6

^1^ The standard deviation of ±0.2 m^2^ g^−1^ was estimated for the BET surface areas calculated from the N_2_ adsorption isotherms.

**Table 2:** Characteristics of the metal nanoparticles (NPs) on the magnetite-based catalysts.

Catalyst	Metal Load ^1^ (wt.%)	Pd Load ^1^ (wt.%)	Pd:Ag ^2^	Pd:Ag (SA-EDS)	Diam. Metal NPs ^4^ (nm)
1% Pd-Fe_3_O_4_	1	1	1:0	-	3.3 ± 1.2
2% Pd-Fe_3_O_4_	2	2	1:0	-	3.4 ± 1.1
3% Pd-Fe_3_O_4_	3	3	1:0	-	4.4 ± 1.6
3% AgPd-Fe_3_O_4_	3	2.4	0.8:0.2	0.8:0.2 ^3^	4.3 ± 1.2

^1^ Pd or Ag-Pd metal load nominally introduced by wet impregnation. ^2^ Atomic ratios employed for wet impregnation. ^3^ An averaged atomic ratio determined by chemical analysis (standard deviation = 0.1) of selected areas employing X-ray energy-dispersive spectroscopy (SA-EDS) coupled to a 200 kV field emission analytical electron microscope employing high-angle annular dark-field (HAADF) imaging in scanning transmission mode (STEM) mode. ^4^ The average diameter of metal nanoparticles (± SD), measured by EsB detector in HRFESEM (1–3% Pd-Fe_3_O_4_) or HAADF imaging in STEM (3% AgPd-Fe_3_O_4_).
